# Segmentation of liver CT images based on weighted medical transformer model

**DOI:** 10.1038/s41598-024-60594-6

**Published:** 2024-04-30

**Authors:** Qun Gu, Hai Zhang, Rui Cai, Si Yi Sui, Rui Wang

**Affiliations:** 1 School of Computer and Communication, Lanzhou University of Technology, Lanzhou, 730050 China; 2 College of Information Technology, Shanghai Jian Qiao University, Shanghai, 201306 China

**Keywords:** Image processing, Machine learning, Predictive medicine

## Abstract

Deep convolutional neural networks have made significant strides in the field of medical image segmentation. Although existing convolutional structures enhance performance by leveraging local image information, they often lose the interdependence information between contexts. Therefore, the article utilizes the multi-attention mechanism of the Transformer structure to more comprehensively express relationships between contexts and introduced the Transformer network architecture into the field of medical image segmentation. Most models based on this Transformer structure typically require large datasets for training. However, in the medical field, the limited size of datasets makes training models with the Transformer structure challenging. To address this, the article propose a Weighted Medical Transformer (WMT) model that imposes low requirements on dataset quantity. The weighting mechanism in the WMT model aims to improve the issue of inaccurate relative positional coding when dealing with small medical datasets. Additionally, a coarse-grained and fine-grained segmentation mechanism is introduced, focusing on both the detailed aspects within image blocks and the boundary information connecting blocks. Experimental results on a liver dataset demonstrate that the model achieves F1 and IoU scores of 88.48% and 79.41%, respectively. Results on the MoNuSeg dataset show comparable high F1 and IoU scores of 79.58% and 66.19%, respectively. The model’s accuracy surpasses that of U-Net++ and U-Net models. Compared to other models, this approach is applicable to scenarios with limited datasets, exhibiting high execution efficiency and accuracy.

## Introduction

In the past few decades, the field of medicine has witnessed a world-renowned technological revolution, in which medical imaging plays an important role in medical diagnosis, disease monitoring, and treatment planning. Du et al.^1^ point out that the main components of medical imaging systems, including ultrasound (US), x-ray, and nuclear magnetic resonance (NMR), are widely used in clinical practice. These emerging medical imaging technologies are able to provide real-time, detailed lesion information to physicians in a non-invasive, non-invasive manner by combining image processing techniques, enabling physicians to more intuitively understand the patient’s condition and improve their grasp of disease trends and physical therapy options. This intuition enables doctors to more accurately formulate treatment plans and improve the accuracy of surgery, which has a positive impact on early disease detection, optimization of treatment plans and monitoring of patient recovery, and also reduces the risk of patients in treatment.

Image segmentation involves a wide range of fields and is one of the most important aspects of medical image analysis, which is mainly used for segmenting and studying the interested parts of medical images to provide a strong technical guarantee for the diagnosis of diseases. Huang et al.^2^ proposed that image segmentation is a fundamental task in computer vision, and its main purpose is to extract meaningful and coherent regions from the image input. In recent years, a wide variety of techniques have been developed in the field of image segmentation, including those based on traditional methods, as well as utilizing convolutional neural networks (CNN)^3^ of the latest image segmentation techniques. With the development of deep learning, more deep learning algorithms^4^ have also been applied to image segmentation tasks.

Initially medical image segmentation used the U-Net model based on convolutional neural networks, which was developed by Ronneberger et al.^5^ A U-shaped network was first proposed, which has a structure similar to the letter “U” and consists of symmetric encoding (downsampling) and decoding (upsampling) paths. Each step in the encoding path contains convolution and pooling operations to gradually reduce the spatial resolution. The decoding path contains upsampling and convolution operations to gradually restore the resolution. At each step of the decoding path, the features at the same level of the previous encoding path are connected to the features at the current decoding level to form a jump connection. Zhou et al.^6^ creatively constructed Unet++, a multi-scale convolutional neural network, by modifying the jump connection layer of Unet. Its uniqueness lies in extracting and fusing features at different levels in a superposition manner by connecting jump connections of all layers. The advantage of this design is that it enhances the network’s ability to perceive and synthesize multi-scale information. Valanarasu et al.^7^ To overcome the complexity of computing affinity, self-attention is decomposed into two self-attention modules. The first module performs autocorrelation on the height axis of the feature map and the second module operates on the width axis, resulting in the Axial Attention U-Net.

Zhang et al.^8^ proposed that although the U-Net model uses a convolutional neural network as its backbone, it still suffers from some inductive bias in local modeling and lacks adequate explanation of long-term image correlation. Due to this limitation, it is not possible to establish global modeling with coherent contextual information, and is less sensitive to segmentation of lesion regions that contain large variations. The Transformer model^9^ has an innate global self-attention advantage, making it excellent in dealing with long-range dependencies and global context modeling. Due to the parallelism of the self-attention mechanism, the training and inference process of the Transformer model can be carried out more efficiently, relative to recurrent neural networks (RNN)^10^ Transformer has better computational efficiency compared to sequential models such as Recurrent Neural Networks (RNN). However, most Transformer-based models usually require large-scale datasets for training, and in the medical field, this increases the challenge of the training process due to the relatively small sample size of the dataset, for this reason, a Weighted Medical Transformer (WMT) model is proposed, which is a weighting mechanism that can improve the relative position coding inaccuracies.

## Results

### Data sets and data preprocessing

The experimental data in this study consists of CT datasets and the MoNuSeg dataset. The dataset includes 420 CT liver image data and 51 MoNuSeg datasets. To facilitate the experimental comparison,200 CT images are subjected to fine-grained segmentation with axial attention mechanism with weights,150 CT images are subjected to coarse-grained segmentation with axial attention mechanism with weights, and the remaining 70 CT images are used as the test set for comparison. Liver tissues have clearer contours compared to other tissues and are less prone to problems such as tissue crossover. Labeled images are generated by labeling in the software. The MoNuSeg dataset utilizes stained tissue images captured at a 40× magnification level. This dataset comprises images from various organs and patients. The training dataset includes 40 images, and the test dataset contains 11 images. The article resized all images to 512 × 512 for experimentation.

### Experimental environment and parameter settings

The experimental environment of this paper is as follows: computer configured with win11 operating system, processor Intel corei7-12700H, graphics card GeForce RTX 3060 GPU computing platform. The programming language is Python and the deep learning framework is PyTorch^11^. The size of the input image is 128 pixels × 128 pixels, the epoch of the model is 400 times, the initial learning rate is 1e-3, the learning rate descent is used, and the binary cross-entropy loss function is used to calculate the loss.

### Evaluation criteria

F1 Score^12^ is the harmonic mean of Precision and Recall, which is used to measure the performance of a binary classification problem. Precision indicates the proportion of samples predicted by the model to be in the positive category that are actually in the positive category. Recall indicates the proportion of samples that are actually positive categories that are predicted by the model to be positive categories. As shown in formula (1):

1 $${\text{F}}1 = 2 \times \frac{{{\text{Precision}} \times {\text{Recall}}}}{{{\text{Precision}} + {\text{Recall}}}}$$

IoU Score^13^ A common metric used to evaluate the performance of image segmentation models. It measures the degree of overlap between the region predicted by the model and the real label. Where Intersection denotes the area of the overlap between the region predicted by the model and the real region, and union denotes the area of the union between the region predicted by the model and the real region. As shown in formula (2):

2 $${\text{Iou}} = \frac{{{\text{Intersection}}}}{{{\text{Union}}}}$$

Acc is also one of the metrics used to evaluate the performance of a classification model. It measures the proportion of samples correctly predicted by the model on the entire dataset. Where “Number of Correct Predictions” is the number of samples correctly predicted by the model and “Total Number of Predictions” is the total number of samples predicted by the model. As shown in formula (3):

3 $${\text{Acc}} = \frac{{\text{Number of Correct Predictions}}}{{\text{Total Number of Predictions}}}$$

## Results and Analysis

### Liver experimental results comparison

**Figure 1:**
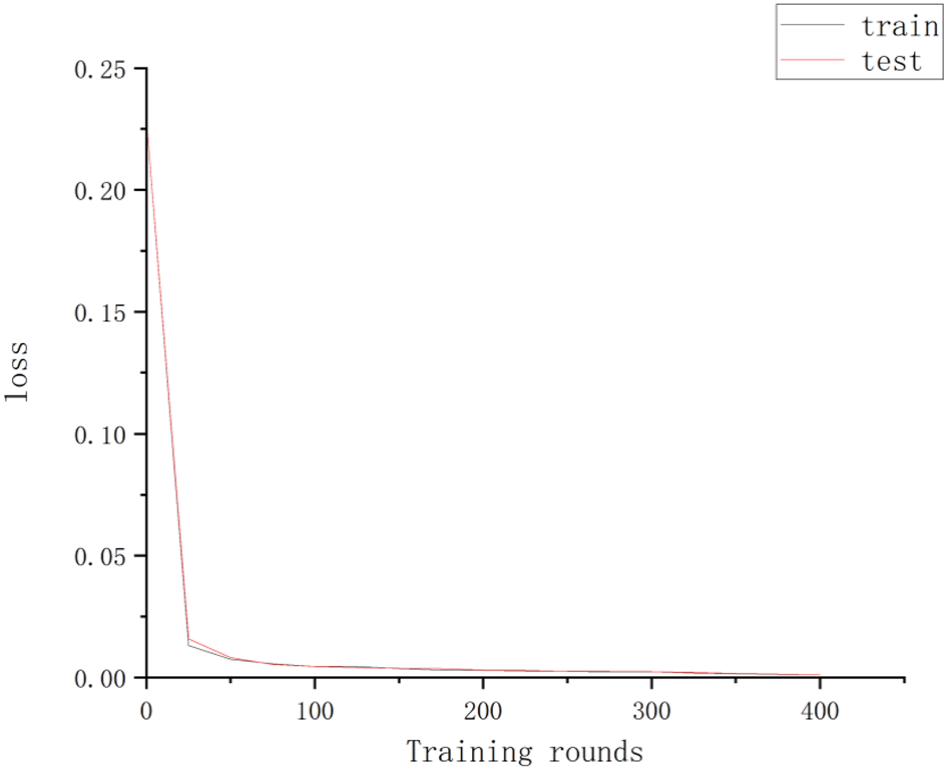
Trend of Loss with the number of training rounds during the training process.

Figure 1 shows the Loss with the number of training rounds (epoch)^14^ It can be seen that the training Loss value decreases as the number of training rounds increases and approaches 0 when the number of training rounds reaches 400; therefore, the number of training rounds in this paper is set to 400.

**Figure 2:**
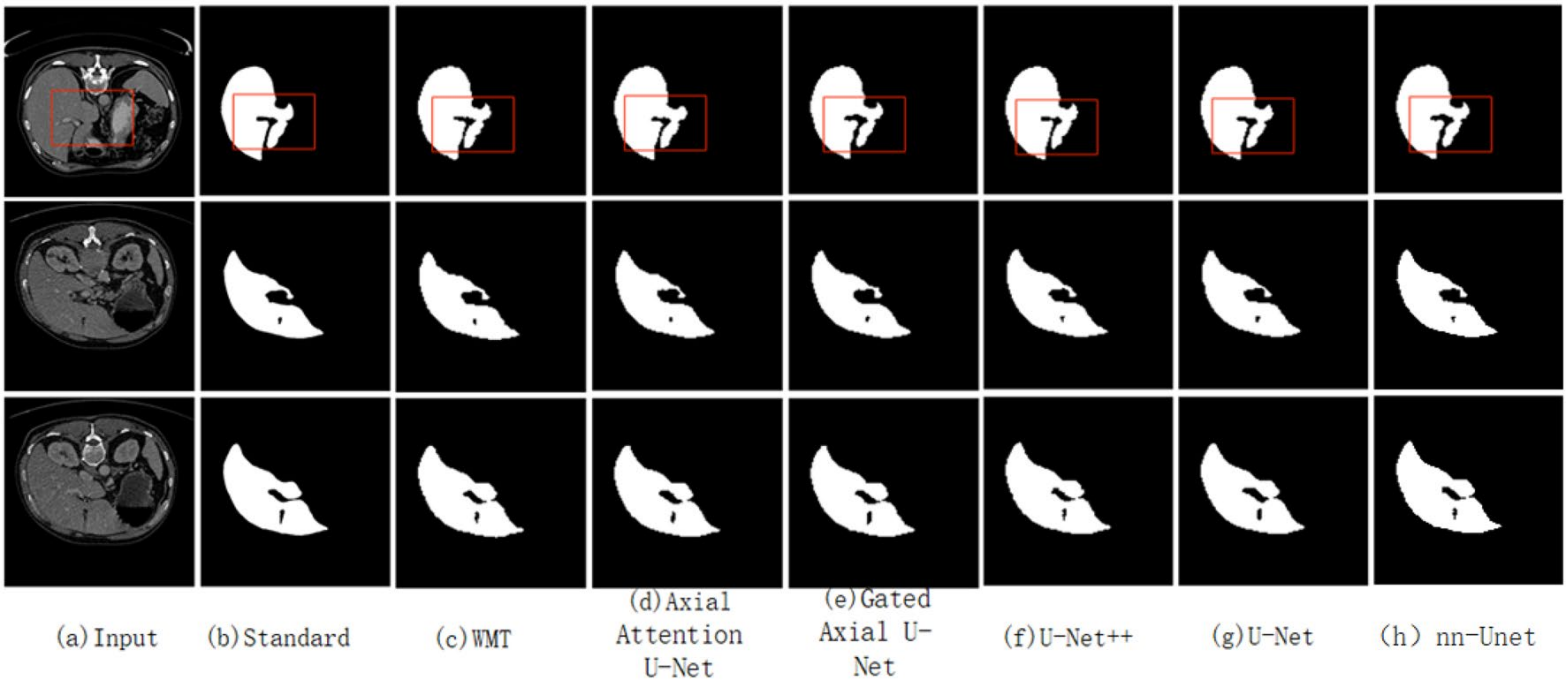
Comparison graph of segmentation performance of different methods.

In this paper, the model performs optimization of liver segmentation accuracy by increasing the weighting mechanism and coarse-grained fine-grained segmentation mechanism in order to optimize the problem of small amount of data in medical images and the problem of intra-block details during segmentation as well as the dependency information between the blocks. For quantitative analysis of different models they are evaluated using F1, IoU and ACC scores, for CT liver dataset the segmentation performance of having axial attention mechanism module is better, this is due to changing the segmentation of the whole 2D image into two axial up to 1D segmentation, as can be seen from the details in the red box the peripheral details of this paper’s model have to be handled better, this is due to the inclusion of coarse and This is due to the addition of coarse-grained and fine-grained mechanisms, the peripheral detail information mainly comes from the coarse-grained segmentation. The internal details mainly come from the addition of fine-grained segmentation, and the method achieves F1 scores and IoU as high as 88.48% and 79.41% on the individual dataset, with a higher model accuracy than many other existing methods. Based on the comparison details in Fig. 2 and the data in Table 1, The method does not require a large dataset as compared to other methods and it has high execution efficiency.

**Table 1:** Comparison of segmentation performance of different methods.

Model	F1 score/%	IoU fraction/%	ACC score/%
U-Net	74.12	58.43	94.36
U-Net++	82.79	75.02	97.02
Axial attention U-Net	75.15	60.97	96.53
Gated axial U-Net	86.91	76.85	97.83
nn-Unet	88.24	79.12	98.15
Weighted medical transformer	88.48	79.41	98.49

### Comparison of experimental results on the MoNuSeg dataset

Table 2 presents comparative data for different segmentation methods on the MoNuSeg dataset. For the MoNuSeg dataset, our model exhibits the highest accuracy, attributed to the combined effects of coarse and fine-grained mechanisms, optimizing cellular edge details.

**Table 2:** Comparison of segmentation performance of different methods.

Model	F1 score/%	IoU fraction/%
U-Net	79.43	65.99
U-Net++	79.49	66.04
Res- U-Net	79.49	66.07
Gated axial U-Net	76.44	62.01
nn-Unet	79.56	66.15
Weighted medical transformer	79.58	66.19

### Loss function

The model uses a binary cross-entropy loss function^15^ to compute the loss, which is generally used to measure the difference between two probability distributions, and in particular to assess the discrepancy between the probabilities predicted by the model and the actual target. As shown in formula (4):

4 $${\mathrm{L}}_{{CE{({\mathrm{p}},\hat{\mathrm{p}})}}} = - \left\{ {\frac{{\mathrm{1}}}{{{\mathrm{wh}}}}\sum\limits_{{{\mathrm{x}} = {\mathrm{0}}}}^{{{\mathrm{w}} - {\mathrm{1}}}} {\sum\limits_{{y = 0}}^{{h - 1}} {\left( {{\mathrm{p}}\left( {{\mathrm{x,y}}} \right){\mathrm{log}}\left( {{\hat{\mathrm{p}}}\left( {{\mathrm{x,y}}} \right)} \right)} \right)} } + \left( {1 - {\mathrm{p}}\left( {{\mathrm{x,y}}} \right)} \right){\mathrm{log}}\left( {{\mathrm{1}} - {\hat{\mathrm{p}}}\left( {{\mathrm{x,y}}} \right)} \right)} \right\}$$

where w and h are the dimensions of the image, $p(x,y)$ corresponds to the pixels in the segmented image. $\hat{p}(x,y)$ represents the prediction output at a specific location, denoted as $(x,y)$. This loss function is used for loss calculation in the Weighted Medical Transformer model.

### Ablation experiments

**Figure 3:**
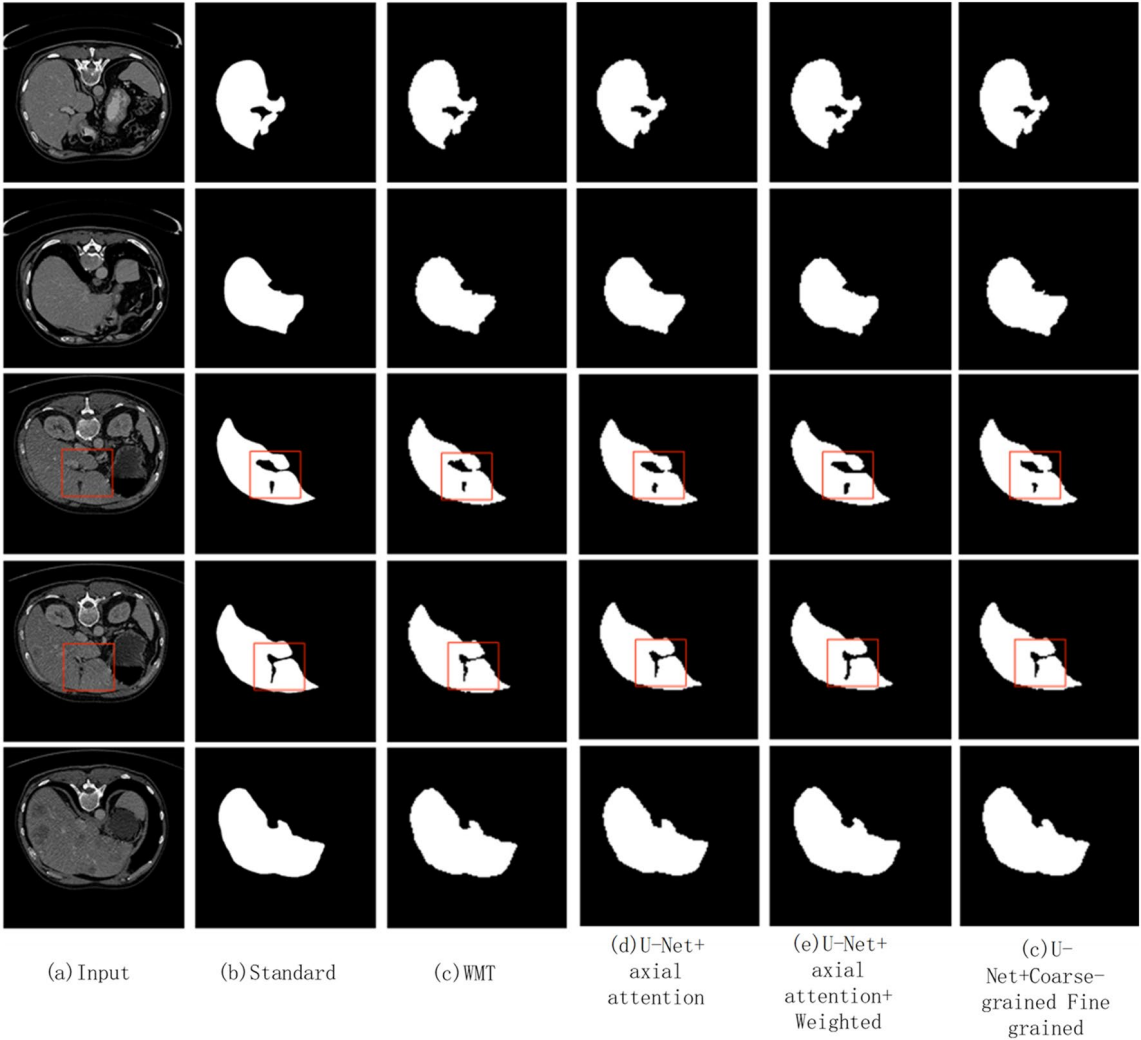
Comparative results of ablation experiments with different modules.

Ablation experiments^16^ are commonly used to assess the robustness of the model and to understand the sensitivity of the model to the input features. When targeting the CT liver image dataset, the red boxes are labeled with segmentation details. In this ablation experiment, each module is added to the U-Net segmentation model and experimental comparisons of each module are performed. The first step replaced all the convolutional layers in the U-Net encoder using the axial attention layer to form the U-Net + axial attention mechanism model, and the second step gave the addition of the weighted axial attention mechanism to form the U-Net + weighted axial attention mechanism, which optimizes the effect of the error in the relative position encoding. The third step adds the coarse-grained and fine-grained fusion methods to U-Net for training to form the U-Net + Fine-grained Coarse-grained Fusion Mechanism model, and finally the improved Weighted Medical Transformer model is trained. According to the details marked in the red box in Fig. 3 and the experimental results of F1 scores, IoU criteria and Acc criteria in Table 3 the model is indeed optimized and improved.

**Table 3:** Comparative results of ablation experiments with different modules table.

Model	F1 score/%	IoU fraction/%	ACC score/%
U-Net	74.12	58.43	94.36
U-Net + Axial Attention	75.15	60.97	96.53
U-Net + weighted axial attention	81.55	68.97	97.31
U-Net + fine-grained coarse-grained fusion mechanism	81.99	69.84	97.63
Weighted medical transformer	88.48	79.41	98.49

## Discussion

This study proposes a model focused on medical image segmentation, incorporating the axial attention mechanism, which significantly enhances the computational efficiency of the model. On top of the axial attention mechanism, a weighted attention mechanism is introduced to regulate the model’s impact on smaller medical datasets. The primary objective of this mechanism is to address the insufficient accuracy of relative positional coding, introducing a weighted mechanism on top of the original relative positional coding to enhance accuracy. Finally, the article conducted coarse-grained and fine-grained segmentation on the images, with fine-grained segmentation focusing more on the details within blocks, while coarse-grained segmentation primarily handles boundary cross-information between blocks. This technology holds the potential to assist physicians in accurate diagnosis, extracting key information, and supporting the determination of optimal treatment plans. In the future, the article plans to integrate the proposed segmentation model into medical image processing platforms, aiming to enhance the accuracy and efficiency of computer-aided diagnosis.

## Methods

The authors declare that all the methods in this article were performed in accordance with the relevant guidelines and regulations in the editorial and publishing policies of Scientific Reports. Lanzhou University of Technology confirmed the experimental approval of CT liver image segmentation. Confirm that all participants and informed consent has been obtained.

### self-attention mechanisms overview

Input a CT feature image $x \in R^{{C_{in} \times H \times W}}$, where the height is H, the weight is W, and the number of channels is C_in_ . After projecting the input, the output of self-attention is computed as ${\text{y}} \in {\text{R}}^{{{\text{C}}_{{{\text{out}}}} \times {\text{H}} \times {\text{W}}}}$. As shown in formula (5):

5 $$y_{ij} = \sum\limits_{h = 1}^{H} {\sum\limits_{w = 1}^{W} {soft\max \left( {q_{ij}^{T} k_{hw} } \right)} } v_{hw}$$

where $q = W_{{Q^{x} }}$, $k = W_{{K^{x} }}$, $v = W_{{V^{x} }}$ are projections computed from input X. ${\text{q}}_{{{\text{ij}}}}$ ,${\text{k}}_{{{\text{ij}}}}$ ,${\text{v}}_{{{\text{ij}}}}$ denote queries, and the keys and values at arbitrary positions are ${\text{i}} \in \{ 1,...,H\}$ and ${\text{j}} \in \{ 1,...,W\}$, respectively. The projection matrices ${\text{W}}_{{\text{Q}}}$, ${\text{W}}_{{\text{K}}}$, ${\text{W}}_{{\text{V}}}$ are learnable and he belongs to ${\text{R}}^{{{\text{C}}_{{{\text{in}}}} \times Cout}}$.

Using the self-attention mechanism^11^ to capture non-local information across the entire feature map may lead to a very high computational cost. The size of the feature map affects computational complexity, and as the dimension of the feature map increases, the computational cost rises sharply. Therefore, directly applying the self-attention mechanism to large-scale images is impractical. The self-attention mechanism does not incorporate positional information when computing non-local contextual information, unlike convolutional layers, which leverage relative positional information through the use of shared-weight filters^12^. In image processing, positional information is often crucial for capturing object structure and relationships, but the self-attention mechanism is insensitive to positional information by default.

### Axial attention mechanisms overview

Local constraints proposed by independent models of self-attention significantly reduce computational cost in visual tasks, making it possible to construct fully self-attentive models. However, such constraints, while helping to maintain local connectivity, limit the range of attentional perceptions to that of a deep convolution with the same kernel size. Furthermore, local self-attention performs within a localized square region with a complexity proportional to the size of the region and introduces another hyperparameter for the trade-off between performance and computational complexity.

**Figure 4:**
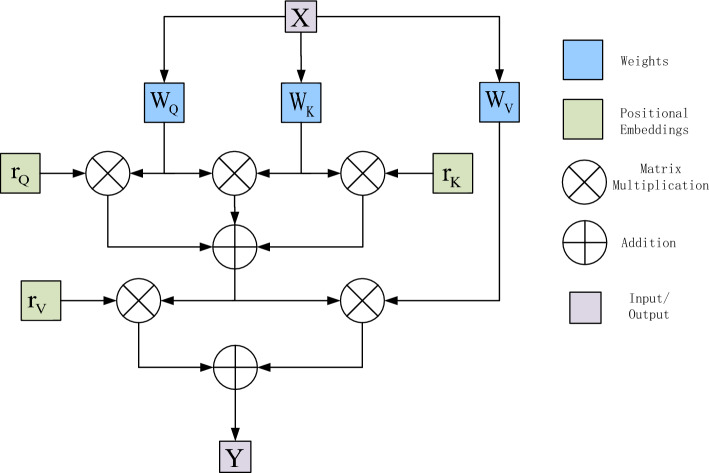
Diagram of axial attention mechanism.

To overcome the computational complexity, the autocorrelation mechanism is decomposed into two autocorrelation modules. The first module performs autocorrelation on the height axis of the feature map and the second module performs autocorrelation on the width axis. This is shown in Fig. 4, This is referred to as axial attention^17^. Therefore, by applying axial attention in both height and width directions, the original self-attention mechanism is successfully modeled and higher computational efficiency is achieved. In order to add positional bias to the computation by in the self-attention mechanism, a positional bias term is added to make sensitive to positional information. Such position bias terms are generally referred to as relative position encodings. These encodings can usually be learned through training and have been shown to be effective in capturing the spatial structure of an image. Using them for all for Q, K, and V. This additional positional bias in Q, K, and V has been shown to capture long-distance interactions with precise positional information. For any given input feature map x, the self-attentive mechanism for updating the position encoding with an axial attention mechanism can be written as shown in (6):

6 $$y_{ij} = \sum\limits_{w = 1}^{W} {soft\max \left( {q_{ij}^{T} k_{iw} + q_{ij}^{T} r_{iw}^{Q} + k_{iw}^{T} r_{iw}^{K} } \right)} \left( {v_{iw} + r_{iw}^{V} } \right)$$

where $r^{Q} ,r^{K} ,r^{V} \in {\text{R}}^{W \times W}$ corresponds to the axial attention model along the width, and similarly, the axial attention model at the height is similar. The aim is to split the 2D image segmentation into two 1D axial segmentations and it does not lose some necessary feature information and it is computationally efficient.

### Axial attention mechanism with weights

**Figure 5:**
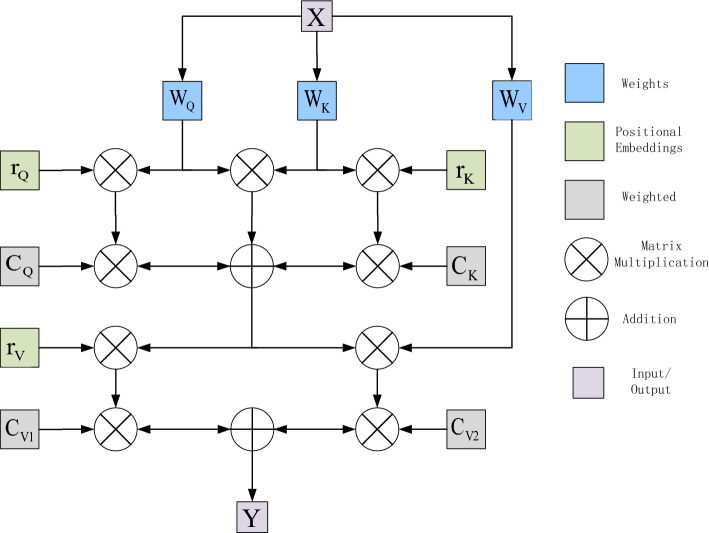
Diagram of axial attention mechanism with weights.

Benefits of the proposed axial attention mechanism for image processing in axial attention. Specifically, the proposed axial attention possesses excellent computational efficiency to efficiently compute non-local contextual information, successfully incorporates positional bias coding into the mechanism, and implements coding of remote interactions in the input feature map. However, the above model is trained on large-scale datasets and the axial attention mechanism is better able to compute the positional bias, but when experimented on small-scale datasets, such as medical image datasets, the positional bias is difficult to learn. Therefore there is some inaccuracy in handling the encoding of long distance interactions. When the relative position coding is not learned accurately enough, applying it to the corresponding Q, K and V may lead to performance degradation. So an improved axial attention block is proposed that can control the effect of positional bias when coding non-local contexts. By adding the weight parameter to the original one, The improvements are shown in Fig. 5, the formula of the axial attention mechanism with weights is expressed as (7):

7 $${\text{y}}_{{{\text{ij}}}} = \sum\limits_{w}^{{\text{W}}} {soft\max } \left( {q_{ij}^{T} k_{iw} + C_{Q} q_{{{\text{ij}}}}^{T} r_{iw}^{Q} + C_{K} K_{iw}^{T} r_{iw}^{K} } \right)\left( {C_{V1} v_{iw} + C_{V2} r_{iw}^{V} } \right)$$

$C_{Q}$, $C_{K}$, $C_{V1}$, $C_{V2}$ Is a learnable parameter, Together, they form a weighting mechanism to regulate the impact of learned relative positional encoding on non-local contextual encoding. in for the position deviation coding accuracy of the part will increase the weight, the accuracy of the part will reduce the weight, weight coefficients in this way to control the position deviation is not accurate enough to bring the impact on the context information.

### Coarse-grained fine-grained segmentation fusion mechanisms

**Figure 6:**
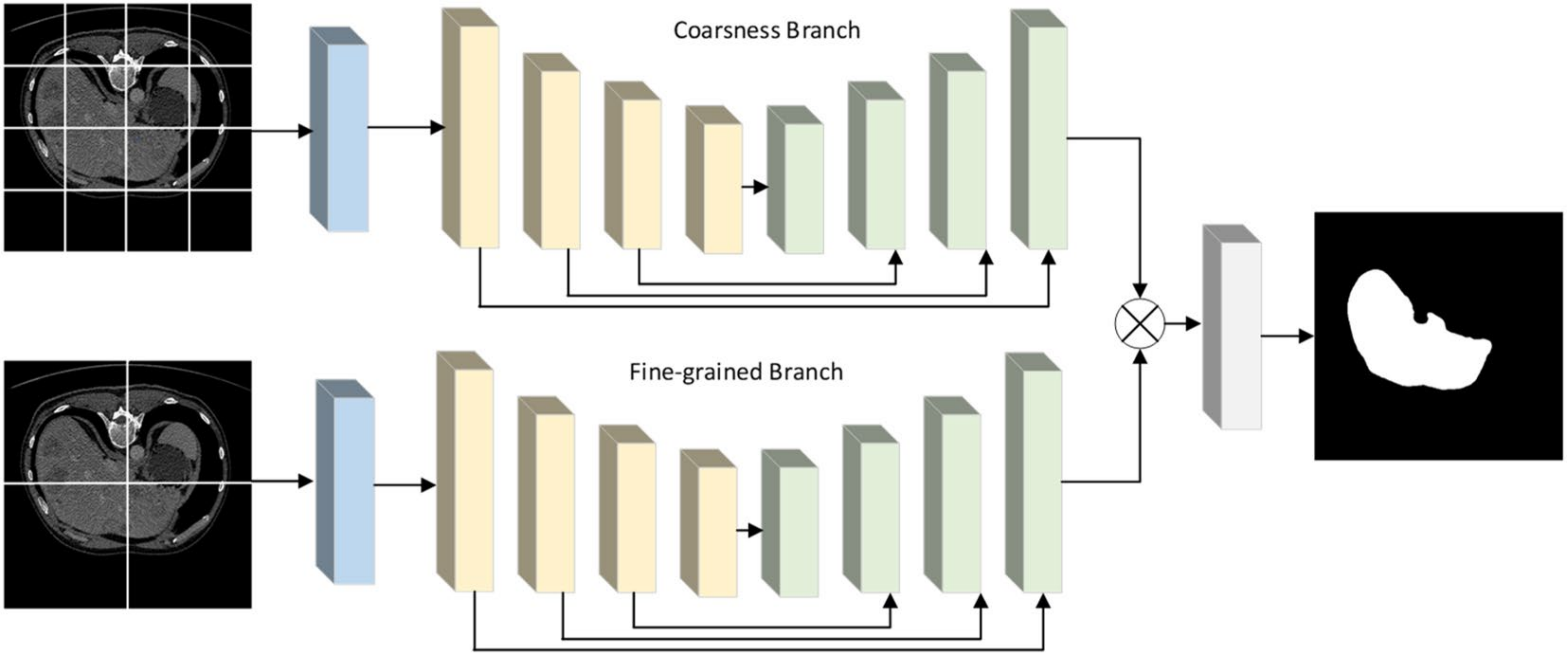
Coarse-grained fine-grained fusion mechanism diagram.

A coarse-grained feature selection module is built, and based on the small blocks selected by the fine-grained module, the semantic and positional relationships between the blocks are mined, so as to obtain coarse-grained diversity features that provide complementary information for the fine-grained blocks. Research experiments show that the Weighted Medical Transformer model on fine-grained faster training, but if all fine-grained training is difficult to complete the image segmentation training task, so that the information between the blocks and their dependencies will be lost, in order to increase the understanding of the information between the blocks and dependencies as well as to improve the accuracy of fine-grained image recognition methods. So this paper proposes a fusion mechanism of coarse-grained segmentation and fine-grained segmentation. In coarse-grained segmentation, the whole image is divided into large chunks, which has the advantage of not ignoring the information between chunks. In fine-grained segmentation, the chunking operation can be set very small, feed-forward operation is performed on each small chunk and the output feature map is resampled according to its position to generate the final output feature map. As shown in Fig. 6, The original image is ${\text{I}}$ Fine-grained segmentation is done using ${\text{I/}}4 \times {\text{I/}}4$ size of 16 chunks and coarse-grained segmentation is done using ${\text{I/}}2 \times {\text{I/}}2$ size of 4 chunks, the output feature maps of coarse-grained segmentation and fine-grained segmentation are summed and passed through the 1 × 1 convolutional layer in order to generate the output segmentation mask. The advantage of this fusion mechanism is that fine-grained segmentation can better capture intra-block information, while coarse-grained segmentation is effective in preserving inter-block boundary information. Using only fine-grained segmentation would neglect the inter-block boundary information.

## Data Availability

This study’s liver dataset can be obtained online at https://github.com/Kaeless/U-Net-liver. This study’s MoNuSeg dataset can be accessed online at https://monuseg.grand-challenge.org/Data/. The data used to support the findings of this study are available from the corresponding author upon request.
